# Molecular and neuronal homology between the olfactory systems of zebrafish and mouse

**DOI:** 10.1038/srep11487

**Published:** 2015-06-25

**Authors:** Luis R. Saraiva, Gaurav Ahuja, Ivan Ivandic, Adnan S. Syed, John C. Marioni, Sigrun I. Korsching, Darren W. Logan

**Affiliations:** 1 Wellcome Trust Sanger Institute, Wellcome Trust Genome Campus, Hinxton-Cambridge, CB10 1SA, United Kingdom; 2 European Bioinformatics Institute (EMBL-EBI), European Molecular Biology Laboratory, Wellcome Trust Genome Campus, Hinxton-Cambridge, CB10 1SD, United Kingdom; 3 Institut für Genetik, Universität zu Köln, Cologne, 50674, Germany

## Abstract

Studies of the two major olfactory organs of rodents, the olfactory mucosa (OM) and the vomeronasal organ (VNO), unraveled the molecular basis of smell in vertebrates. However, some vertebrates lack a VNO. Here we generated and analyzed the olfactory transcriptome of the zebrafish and compared it to the olfactory transcriptomes of mouse to investigate the evolutionary and molecular relationship between single and dual olfactory systems. Our analyses revealed a high degree of molecular conservation, with orthologs of mouse olfactory cell-specific markers and all but one of their chemosensory receptor classes expressed in the single zebrafish olfactory organ. Zebrafish chemosensory receptor genes are expressed across a large dynamic range and their RNA abundance correlates positively with the number of neurons expressing that RNA. Thus we estimate the relative proportions of neuronal sub-types expressing different chemosensory receptors. Receptor repertoire size drives the absolute abundance of different classes of neurons, but we find similar underlying patterns in both species. Finally, we identified novel marker genes that characterize rare neuronal populations in both mouse and zebrafish. In sum, we find that the molecular and cellular mechanisms underpinning olfaction in teleosts and mammals are similar despite 430 million years of evolutionary divergence.

Most mammals have two major olfactory organs: the olfactory mucosa (OM) and the vomeronasal organ (VNO) (reviewed in^1^), which sense odorants and social olfactory cues, and can give rise to changes in behavior or physiology^2^,^3^,^4^. Due to an absence or evolutionary loss of the VNO, some vertebrate lineages – like teleost fish and higher primates, respectively – sense their olfactory environment via a single functional olfactory organ, the OM. Unfortunately the basic molecular and cellular mechanisms of single organ olfaction remain largely unexplored, as most of our knowledge about olfaction and olfactory-mediated behavior in vertebrates arise from studies in mice, which have both an OM and a VNO.

Zebrafish, a teleost fish, has recently emerged as a model system for studying the molecular genetics of olfaction in vertebrates. Despite having diverged from tetrapods ~430 million years ago^5^, the basic organization of the olfactory system in both lineages is thought to be conserved (reviewed in^6^). This idea arose primarily from morphological comparisons and/or by examining the expression of small pools of candidate genes identified from studies of the mouse olfactory system^7^,^8^,^9^,^10^,^11^,^12^. However, the large size of three canonical zebrafish chemosensory gene families (*or*, *taar*, and *olfC/V2r*), combined with the high degree of nucleotide identity among their members, make it very difficult to perform comprehensive expression analysis by *in-situ* hybridization (ISH), quantitative RT-PCR, or even microarray. Consequently, the gene expression landscape of the zebrafish olfactory system is largely unknown, and thus the global gene expression pattern and molecular relationship between the fish and the mouse olfactory systems is not well resolved.

Here we performed RNA sequencing (RNA-seq) to characterize the transcriptome of the zebrafish olfactory system. We observed differences in the expression profile of chemosensory receptor genes, with some being expressed at very high and others at very low levels. In addition, we found a strong positive correlation between the RNA-seq expression values and the number of neurons in the OM expressing a given receptor. This revealed that the recurrent gains/losses of chemosensory receptors during evolution were accompanied by simultaneous increases/decreases in the representation of each neuronal class. The molecular conservation between the zebrafish and mouse olfactory systems goes beyond the receptor level, with orthologs of mouse OM- and VNO-specific genes being expressed at high levels in the zebrafish OM. Finally, we developed a strategy to identify novel cell types with putative chemosensory functions in both mouse and zebrafish. Together, our results show that the basic molecular and cellular mechanisms underlying olfaction in mammals were already present prior to the divergence of tetrapods from teleosts.

## Results

### Zebrafish olfactory transcriptome

**Figure 1:**
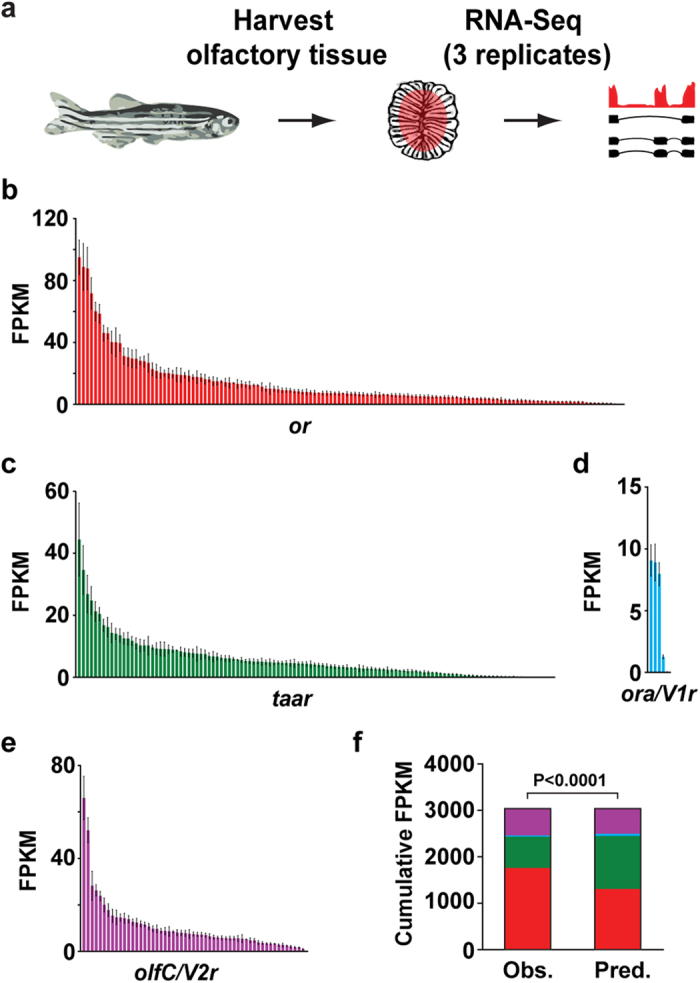
Expression distribution of the chemosensory receptors in the zebrafish OM. (**a**) RNA-Seq experimental strategy. After dissecting the olfactory mucosa (OM) of adult male zebrafish, RNA was extracted, cDNA generated, and libraries for deep-sequencing amplified. The libraries were then sequenced on a HiSeq2500 with 100 bp paired-end reads. (**b**–**e**) Distribution of mean FPKM expression values for each of the *or* (red), *taar* (green), *ora/V1r* (blue) and *olfC/V2r* (purple) genes in the zebrafish OM. Genes are displayed in descending order of their mean expression values. The error bars represent the standard error of the mean (SEM) from 3 sample replicates (each containing pooled OM from 4–5 adult male zebrafish). (**f**) The observed relative expression of each chemosensory receptor gene family differs from a predicted model where each receptor gene is expressed equally (χ^2^, P < 0.0001). *or* genes in red, *taar* genes in green, *ora/V1r* genes in blue, and *olfC/V2r* genes in purple.

We used RNA-seq to profile the polyadenylated RNA fraction of the whole OM from adult male zebrafish. Libraries generated from 3 sample replicates (each containing pooled OM from 4-5 adult male zebrafish) yielded an average of 36.1 ± 11.2 million (mean ± standard error) 100 bp, paired-end Illumina HiSeq2500 reads (Fig. 1, Supplementary Table S1). To analyze our data, we used iRAP, a computational pipeline that integrates existing tools for filtering and mapping reads, quantifying expression and testing for differential expression^13^. On average, 70.19 ± 0.48% of the total reads mapped uniquely to the annotated zebrafish genome (Ensembl Zv 9.0, release 73; Supplementary Table S1). To estimate gene-specific expression levels we calculated FPKM values (fragments per kilobase of transcript model per million reads), by counting and normalizing the gene-specific uniquely mapped reads to the gene length, and sequencing depth (see Methods).

A comparison between the three sample replicates revealed extremely low variability levels, as demonstrated by the high Spearman correlation coefficients (~0.98, *P* < 0.0001, Supplementary Fig. S1a). Gene expression levels follow a bimodal distribution corresponding to low-expressed (LE) and high-expressed (HE) genes, a characteristic of RNA-seq data from tissues or large cell populations^14^. Since low-expressed genes are enriched in non-functional mRNAs and lack active chromatin marks and correlative protein data^14^, we decided to focus our analysis on the genes that have a ≥25% chance of being within the HE distribution. We find 21549 (76.8%) genes that fall in this distribution (Supplementary Fig. S1b). Moderate-highly expressed genes (≥1 FPKM) represent 66% of the total number expressed (Supplementary Data S1). The 200 most abundant genes account for 46% of the total cumulative FPKM.

To explore the function of the expressed genes we performed a Gene Ontology (GO) analysis (Supplementary Fig. S1c). Under the “Molecular Function” category, the *Ion binding*, *Nucleotide binding*, and *Nucleic acid binding* account for 53% of the classified genes, and within the “Cellular Component” category, 64.9% of all the classified genes belong to the *Membrane*, *Nucleus*, or *Macromolecular complex* classes (Supplementary Fig. S1c). In the “Biological Processes” category, *Metabolic process, Biological regulation* and *Response to stimulus* account for a combined 55.5% of all classified genes. While the terms *Metabolic process* (26.79%) and *Biological regulation* (16.54%) are mainly associated with genes involved in housekeeping functions, the term *Response to stimulus* (12.16%) is strongly associated with genes with chemosensory functions (Supplementary Fig. S1c, and data not shown). Together, these results suggest that approximately half of the classified genes have either housekeeping or chemosensory functions.

### The zebrafish chemosensory receptors are differentially expressed

We and others have previously identified 133 olfactory receptors (*or*)^9^,^15^, 112 trace-amine associated receptors (*taar*)^7^, 6 olfactory receptor type A/ vomeronasal receptor type 1 (*ora/V1r*)^8^, and 54 olfactory receptor type C/ vomeronasal receptor type 2 genes (*olfC/V2r*)^10^ in zebrafish. The most recent zebrafish genome assembly contains a total of 314 annotated chemosensory receptors: 135 *or*, 118 *taar*, 5 *ora/V1r*, and 56 *olfC/V2r* genes. We used only uniquely mapped reads to analyze the expression distribution of the chemosensory receptors in the zebrafish OM (see Methods). Within each family, a large dynamic range of expression levels was observed (Fig. 1b–e, Supplementary Data S2). This distribution deviates significantly from a model where each receptor is expressed at the same level (χ^2^, *P* < 0.0001, Fig. 1f). We find 23 *or*, 6 *taar* and 6 *olfC/V2r* genes with expression values greater than 20 FPKM, which account for 55.9%, 25.5% and 37.4% of the cumulative expression values of their respective gene families. In contrast, the majority (80%) of the *ora/V1r* genes display similar levels of expression. We found evidence of expression for all annotated *or* and *ora/V1r* genes, but 3 *taar* genes (*taar20p, taar12a*, and *si:ch211-238p8.35*) and 1 *olfC* gene (*v2rh25p*) had no mapped reads to them in any replicate (Supplementary Data S2).

Most chemosensory receptors are located in genomic clusters. We thus asked whether the chromosomal location, or location within a cluster, influences the receptor expression levels, but observed no obvious patterns (Supplementary Fig. S2). These results show that in the zebrafish OM, the chemosensory receptor expression profile is differential but stereotypic, with different receptors reproducibly expressed at different levels between replicates.

### RNA-seq expression levels correlate with the number of OSNs expressing chemosensory receptors

**Figure 2:**
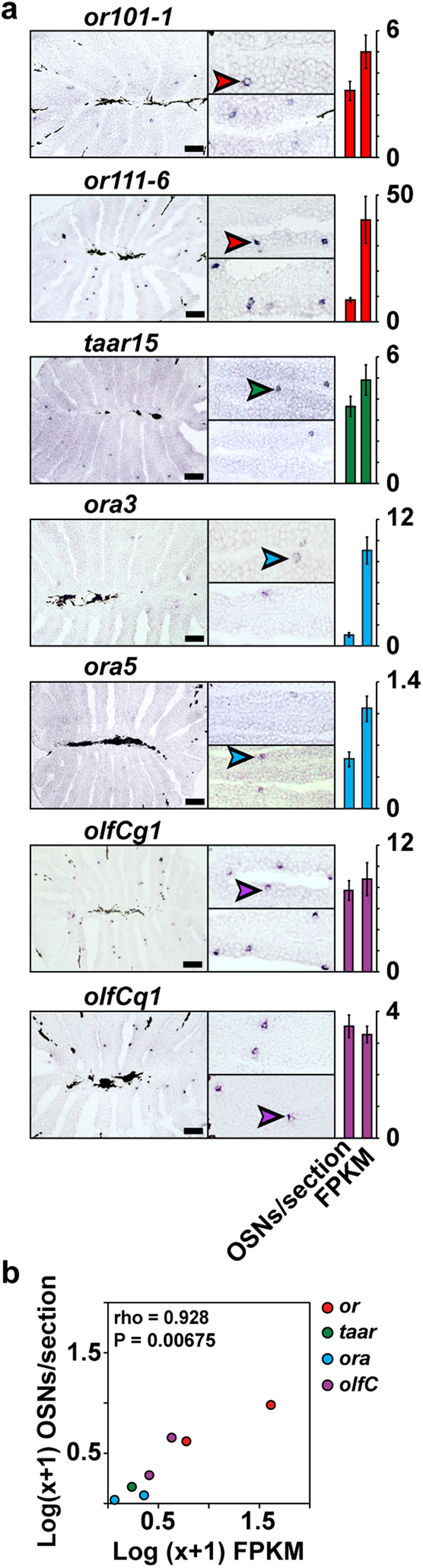
Chemosensory receptor gene expression correlates with number of neurons in zebrafish OM. (**a**) Cryosections of adult zebrafish OM were hybridized with cRNA probes for *or101-1, or111-6*, *taar15*, *ora3*, *ora5*, *olfCg1*, *olfCq1*. Representative micrographs show expression in complete sections (left panel), and single lamella (right panels). The hybridization signal was observed in sparse cells within the sensory region of the OM. Arrowheads point to labeled OSNs. To the right of each micrograph a bar graph shows number of labeled OSNs/section (mean +/− SEM, n = 47–72), and the corresponding RNA-seq expression values (mean +/− SEM, n = 3). (**b**) Spearman correlation of FPKM values and OSN density determined by ISH. Scale bars, 50 μm.

Consistent with the ‘one receptor-one neuron’ rule^16^,^17^, we hypothesized that our RNA-seq chemosensory receptor expression profile may reflect variance in the number of olfactory neurons expressing different receptors in the zebrafish OM^18^. To investigate this, we performed *in-situ* hybridization (ISH) for two genes from each of the chemosensory receptor families: *or101-1, or111-6*, *taar15, taar19l*, *ora3, ora5*, *olfCg1,* and *olfCq1* (*vrh14*). The ISH experiments showed in all but one case (*taar19l*) the sparse expression pattern characteristic of chemosensory receptors (Fig. 2a and Supplementary Fig. S3a). *taar19l* is a member of a large subfamily of very closely related genes (sharing >85% identity at the nucleotide level with 20 other *taar* receptors), for which extensive cross-reactivity may be expected^19^; thus it was not included in our downstream analysis. For the remaining seven genes we find a very strong correlation between the RNA-seq FPKM values and the number of OSNs expressing the given receptor (Spearman rho = 0.928, *P* = 0.00675, Fig. 2b). To cover an even wider range of expression, we retrieved previously published ISH data for ten additional *or* genes: *or102-1, or103-1, or111-10, or111-7, or111-5, or111-3, or111-2, or111-1, or107-1, or19-2*^20^. Notably, we still find a strong correlation between the ISH expression measurements and their respective FPKM values when this second, independent dataset is included (Spearman rho = 0.745, *P* = 0.00059, Supplementary Fig. S3b). Taken together, and consistent with conclusions drawn from similar analyses in the mouse OM^18^, these results suggest that RNA expression levels of ORs are robust predictors of the number of OSNs that express a given chemosensory receptor.

### The zebrafish and mouse share biases in chemosensory neuron repertoires

Given that receptor expression levels vary significantly (Fig. 1f), but correlate well with the number of sensory neurons that express that receptor (Fig. 2a,b), we can use FPKM values to estimate the relative proportion of each class of olfactory neuron (those expressing different families of chemosensory receptor) in an olfactory organ.

**Figure 3:**
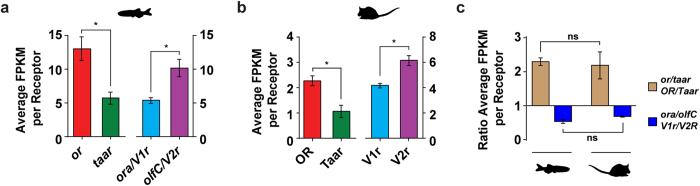
Shared biases in the zebrafish and mouse sensory neuron repertoires. Average expression values per receptor gene in zebrafish (**a**) and mouse (**b**), for each of the chemosensory receptor gene families (mean +/− SEM, n = 3). (**c**) Ratio of the average expression values per receptor gene of *or*/*OR:taar/Taar* and *ora/V1r:olfC/V2r* in zebrafish and mouse.

First we asked if there is a bias in the expression levels between the chemosensory receptor families. In zebrafish, *or* genes account for 43% (135 genes) of the total chemoreceptor gene repertoire, with *taar*, *ora/V1r*, and *olfC/V2r* genes accounting for the remaining 37.6% (118 genes), 1.6% (5 genes), and 17.8% (56 genes), respectively (Supplementary Fig. S4a). If the members of each receptor gene family had an equal probability of being expressed in the OM, the relative contributions of each family to the total chemosensory gene repertoire and to the cumulative expression level should correspond to these percentages. However, the *or*, *taar*, *ora/V1r* and *olfC/V2r* families contribute 57.8% (1757.0 ± 236.2 FPKM), 22.2% (675.7 ± 104.0 FPKM), 0.9% (27.2 ± 2.2 FPKM) and 19.1% (579.5 ± 73.9 FPKM) respectively (Supplementary Fig. S4b), representing a significant difference (Fig. 1f, χ2, *P* < 0.0001). After normalization for receptor gene number, *or* and *olfC* expressing neurons are significantly enriched in the zebrafish olfactory system relative to those expressing *taar* and *ora* receptor genes (Fig. 3a).

The chemosensory gene repertoire is largely species-specific, shaped by the nature of chemosensory information necessary for survival in each species’ niche^21^,^22^. For example, during the water to land transition of vertebrates, the ratio of intact *V1r* to *V2r* genes increased ~50-fold^23^. How do changes in the intact chemosensory receptor gene number influence their representation in the nose? To address this question we started by comparing the chemoreceptor expression distributions in the zebrafish OM with the equivalent distributions in the mouse OM and VNO. Similar to zebrafish, after adjustment for gene number the distribution of mouse OSNs expressing *ORs* is enriched compared to *Taars*, and *V2r* expressing neurons are enriched relative to those expressing *V1r* receptors (Fig. 3b, and Supplementary Fig. S4c–e). Together these results show that, despite a large dynamic range of receptor expression *within* each family (Fig. 1), after adjusting for gene number the relative neuronal representation *between* each class of chemosensory neuron differs in a consistent manner between mouse and zebrafish. In other words, the absolute neuronal representation of each class is scaled by large differences in receptor gene repertoire, but the underlying logic is similar in two species separated by ~430 million years of vertebrate evolution. Future studies including many species from different evolutionary branches will show whether this similarity is a consequence of evolutionary conservation or convergence.

### Global comparison of the olfactory transcriptomes of zebrafish and mouse

Recently, we and others have reported the existence of tetrapod VNO genetic components in the teleost fish OM, which suggests the existence of an ancestral “vomeronasal” pathway in the most recent common ancestor (MRCA) of fish and mammals^8^,^24^,^25^. Because these studies focused on a small number of VNO-specific genes, the overall evolutionary relationship between the fish and mammalian olfactory systems still remains unclear.

**Figure 4:**
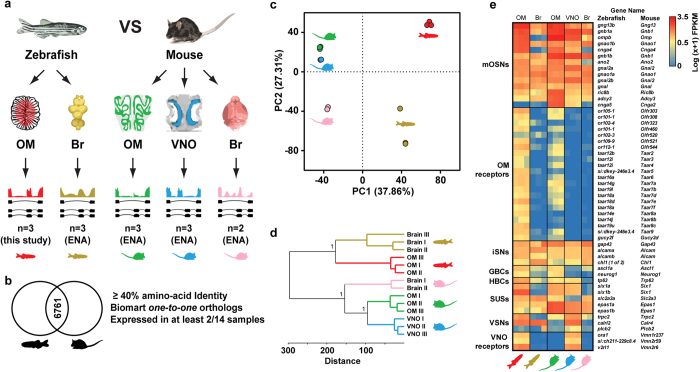
Comparison of the olfactory transcriptomes of zebrafish and mouse. (**a**) The transcriptomes from the zebrafish brain (Br, olive), and mouse OM (green), VNO (blue) and brain (Br, pink) were compared to the transcriptome of the zebrafish OM (red). (**b**) Venn diagram indicating the Biomart 6761 orthologous gene pairs between zebrafish and mouse used in downstream analysis. Triage steps used are indicated on the right. (**c**) Principal component analysis (PCA) of the tissue RNA-seq expression levels for the 6761 Biomart ortholog pairs. Percentages of the variance explained by the principal components (PCs) are indicated in parentheses. PC1 separates species, while PC2 separates tissues. (**d**) Hierarchical clustering analysis (HC) of the tissue expression profiles for the 6761 Biomart ortholog pairs. Bootstrap values (100 boostraps, 1 represents >0.999) for the 3 major nodes are indicated. (**e**) Heatmap of the expression pattern of mammalian olfactory cell-specific markers and corresponding zebrafish orthologs across all tissues analyzed. There is conservation of expression of both mouse OM and VNO specific markers in the zebrafish OM. RNA expression levels are represented on a log scale (0 < low ≤ 0.63, 0.63 < moderate ≤ 1.8, high > 1.82). mOSNs: mature OSNs, iSNs: immature OSNs, GBCs: globose basal cells, HBCs: horizontal basal cells, SUSs: sustentacular cells, VSNs: vomeronasal sensory neurons.

To explore this we compared the full transcriptome of the zebrafish OM to that from mouse OM and VNO (Fig. 4a and Supplementary Fig. S5b,c). As out-groups we included the transcriptomes of the mouse and zebrafish brains (Fig. 4a and S5a–d). To enable a direct cross-species comparison we used Biomart to establish orthology relationships between the mouse and zebrafish genes^26^. We focused our analysis on high confidence ‘one-to-one’ orthologs that have amino-acid identity values of at least 40%, and that are expressed in at least two (of the 14) tissue replicates across all tissues (Fig. 4b). Subsequently, we applied principal component analysis (PCA), and hierarchical clustering (HC) to the remaining 6761 ortholog gene pairs. Unexpectedly, the samples separated first by species (PC1, 37.86% of the variance), and only secondarily by tissue types (PC2, 27.31% of the variance) (Fig. 4c). The HC analysis further supports these results, with the expression patterns of mouse tissues being more closely related, than functionally similar tissues between species (Fig. 4d).

In mammals the sensory neuro-epithelium of the olfactory mucosa is a pseudo-stratified epithelium composed of multiple cell types, including: mature OSNs and VSNs (mOSNs and mVSNs, respectively), immature sensory neurons (iSNs), globose basal cells (GBCs), horizontal basal cells (HBCs), and sustentacular cells (SUSs)^27^,^28^,^29^,^30^. While analogous cell types have been found in the zebrafish OM, the stratification of the olfactory neuro-epithelium is inconspicuous, as different cell types do not segregate into layers^31^. To explore this further, we compared the expression profiles of molecular markers for different cell types in the zebrafish and the mouse olfactory systems (Fig. 4e). Of the 29 cell-specific markers expressed in the mouse OM and/or VNO, 28 zebrafish orthologs are expressed, and only one (*cnga5*) is not expressed in the zebrafish OM (Fig. 4e). As expected, when we applied PCA and HC to the expression levels of these 29 cell-specific markers, we find that the samples separated first by tissue (PC1, 41.45% of the variance), and only secondarily by species (PC2, 28.73% of the variance) (Supplementary Fig. S6).

Together this demonstrates that global gene expression patterns between mouse and zebrafish olfactory organs are not highly correlated, but expression profiles of genes known to be specifically involved in olfactory perception appear conserved. Moreover such conservation extends beyond the level of the mature OSNs and their receptors to the other cell types present in the zebrafish OM.

### Reciprocal identification of novel genes that sub-classify neurons

Recent studies have identified a small number of additional, non-canonical chemosensory receptors expressed in neurons in the OM and VNO of mice. One, *Gucy2d* (GC-D), is a membrane guanylate cyclase expressed in the mouse OM^32^,^33^,^34^,^35^,^36^,^37^. The others are formyl-peptide receptors (*Fpr-rs1*, *Fpr-rs3*, *Fpr-rs4*, *Fpr-rs6*, and *Fpr-rs7*), which detect disease/inflammation-related ligands via the VNO^38^,^39^,^40^. Although homologous genes have been identified in zebrafish, it remains unclear whether they serve a similar chemosensory function.

**Figure 5:**
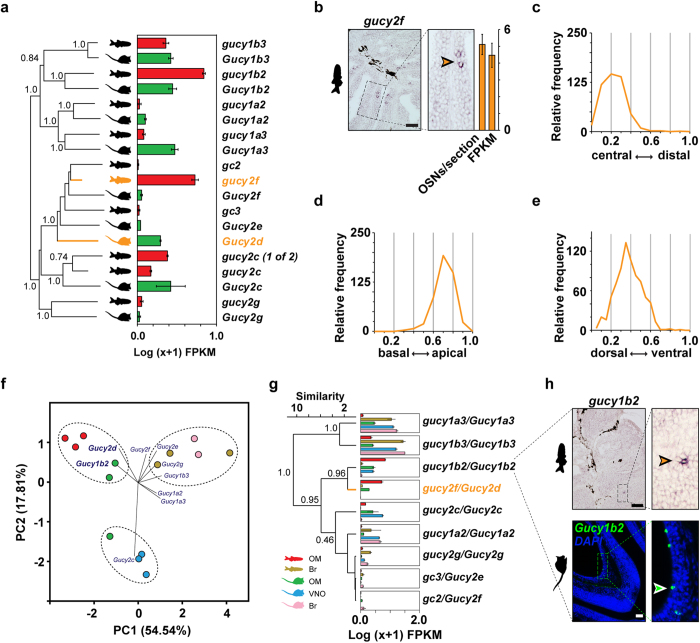
Novel genes with putative olfactory functions in the mouse and zebrafish OM. (**a**) Phylogenetic tree of all guanylate cyclase genes of mouse (*Gucy*) and zebrafish (*gucy*). Bootstrap values (100 bootstraps, 1 represents >0.999) for the major nodes are indicated. Orange branches represent the mouse *Gucy2d* (GC-D), and its likely zebrafish ortholog, *gucy2f*. To the right of the tree, a bar graph indicates the RNA-seq expression values (mean +/− SEM, n = 3) for the *gucy/Gucy* genes in the OM. (**b**) Cryosections of adult zebrafish OM hybridized with a cRNA probe for *gucy2f*. Micrographs show expression in a complete section (left panel), and single lamella (right panels). The arrowhead points to a single labeled OSN. To the right of the micrograph a bar graph shows number of labeled OSNs/section (mean +/− SEM, 47 ≤ n ≤ 72), and the corresponding RNA-seq expression values (mean +/− SEM, n = 3). (**c**–**e**) Three spatial parameters were quantified for the *gucy2f*-positive neuron population, shown as histograms. (**f**) PCA of the tissue RNA-seq levels for nine Biomart ortholog *gucy/Gucy* gene pairs. Percentages of the variance explained by the PCs are indicated in parentheses. Functionally related tissues cluster together in 3 major groups. The central biplot shows a projection of the *Gucy* genes onto the scattergram. *Gucy1b2* and *Gucy2d* drive the clustering of most of the OM samples. (**g**) Hierarchical clustering analysis (HC) of the tissue expression profiles for nine Biomart orthologous *gucy/Gucy* gene pairs. Bootstrap values (100 bootstraps, 1 represents >0.999) for the major nodes are indicated. To the right of each branch, bar graphs showing the RNA-seq expression values (mean +/− SEM, n = 2-3) for the *gucy/Gucy* genes in the mouse and zebrafish olfactory organs, and brain. (**h**) Cryosections of adult zebrafish and mouse OM hybridized with cRNA probes for *gucy1b2* and *Gucy1b2*, respectively. The hybridization signals are sparsely distributed within the sensory region of the OM, as is typical for chemosensory receptor genes. Arrowheads point to single labeled OSNs. Scale bars, 50 μm.

To investigate this, we started by reconstructing the phylogeny of these gene families. We found three zebrafish genes that cluster within a clade containing mouse *Gucy2d* (*gucy2f, gc2*, and *gc3).* Although none emerge as a clear direct ortholog, we find that only *gucy2f* is expressed in the zebrafish OM (Fig. 5a). Next we performed ISH and detected strong *gucy2f* expression in a small subset of OSNs scattered throughout the OM (Fig. 5b). These cells were restricted to the inner, sensory surface, and occurred in low frequency – less than one labeled cell per lamella – typical for monogenic expression of chemosensory receptor genes^41^. A characteristic of canonical OR genes is that their expression is centered within sub-regions of the olfactory epithelium. Quantitative evaluation of coordinates of *gucy2f*-expressing cells showed a preference for central localization (Fig. 5c), albeit not as extreme as the distribution previously observed for the odorant receptor, *or112-1* (*zor6*)^41^. Within each lamella, cells expressing *gucy2f* are preferentially located apically, near the lumen (laminar height parameter, Fig. 5d). For the third spatial parameter, vertical height (z-axis), the cells are enriched in the more dorsal regions of the olfactory tissue (Fig. 5e). Thus, similar to *or* genes, *gucy2f*-expressing cells have a specific location along the three spatial axes. Together these data suggest that *gucy2f* is expressed in specific zebrafish OSNs, comparable to the chemosensory role mediated by mouse *Gucy2d*.

These results raise the possibility that specific expression of other orthologous genes in the olfactory systems of both species could also be indicative of putative olfactory functions. To test this, we applied PCA to the tissue RNA expression levels for the nine Biomart orthologous *gucy/Gucy* gene pairs between zebrafish and mouse (Fig. 5f). Principal components 1 (PC1) and 2 (PC2), explain the majority (72.35%) of the variance in these data. Interestingly, we find that functionally related tissue samples cluster together: one group is specific for the mouse and zebrafish OM, one for the mouse VNO, and one for the zebrafish and mouse brains. Projecting the *Gucy* genes onto the scattergram revealed that two orthologous gene pairs are driving the clustering of the OM samples: *gucy2f/Gucy2d* and *gucy1b2/Gucy1b2* (Fig. 5f). A HC analysis of the same data matrix revealed very similar results, with *gucy2f/Gucy2d and gucy1b2/Gucy1b2* clustering together with high bootstrap support, consistent with their specific expression in the mouse and zebrafish OM (Fig. 5g). *gucy1b2* and *Gucy1b2* are expressed at even higher levels in the zebrafish and mouse OM than *gucy2f* and *Gucy2d*, respectively, the latter being a known chemosensory receptor in mouse (Fig. 5g). We therefore hypothesized that *gucy1b2/Gucy1b2* may serve a similar chemosensory role in the zebrafish and mouse OM. We performed ISH in cryosections of adult zebrafish and mouse OM, with cRNA probes for *gucy1b2* and *Gucy1b2*. In both cases the probes labelled a subset of OSNs scattered in the OM (Fig. 5h). By counting labeled cells, we estimate that mouse OM contains at least 2800 *Gucy1b2*+ cells (89.1 ± 10.214 cells/section, mean ± sem) and zebrafish has at least 250 *gucy1b2*+ cells (0.5 ± 0.038 cells/section, mean ± sem). This is within the range of counts we found for single chemosensory receptor labeled neurons (Fig. 2).

**Figure 6:**
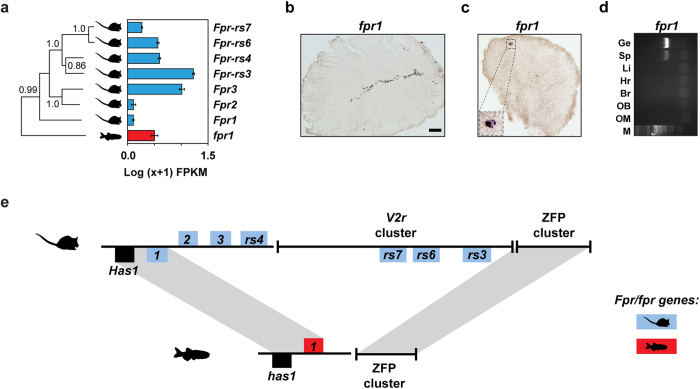
Phylogeny, expression, and synteny of the *fpr/Fpr* gene family in the zebrafish and mouse olfactory systems (**a**) Phylogenetic tree of all formyl peptide receptors of mouse (*Fpr*) and zebrafish (*fpr*). Bootstrap values (100 bootstraps, 1 represents >0.999) for the major nodes are indicated. To the right of the phylogenetic tree a bar graph shows the RNA-seq expression values (mean +/− SEM, n = 3) for the *Fprs* in the mouse VNO (blue bars), and the *fprs* in the zebrafish OM (red bars). Cryosections of adult zebrafish OM (**b**) and spleen (**c**) hybridized with a cRNA probe for *fpr1*. No expression was observed in the OM. (**c**) The lower left corner magnified panel in (**c**) shows a cell (possibly a macrophage) expressing *fpr1.* (**d**) Expression of *fpr1* mRNA detected by RT-PCR in the spleen. PCR amplifications were performed by using gene-specific primers. OM, olfactory mucosa; OB, olfactory bulb; Br, brain; H, heart; L, liver; Sp, spleen; Ge, genomic DNA. Scale bars, 50 μm. (**e**) Genomic synteny between the zebrafish and mouse *fpr/Fpr* loci. In both species the *fpr/Fpr* genes are flanked by the *has1*/*Has1* gene and a zinc-finger protein (ZFP) gene cluster. The mouse-specific *Fpr* genes are located within, and immediately adjacent to, a *V2r* cluster.

In contrast to the high orthology between zebrafish and mouse *Gucy* genes, we found only one *Fpr* gene in the most recent zebrafish genome assembly, *fpr1* (Fig. 6a). Although *fpr1* shows a moderate expression value in the RNA-seq data, we did not observe any OSNs or other cells expressing *fpr1* in the OM (Fig. 6b) and therefore hypothesized that the RNA-seq expression has its origin in the neutrophils and monocytes present in the organ’s blood supply. We performed ISH and reverse transcriptase PCR (RT-PCR) in the zebrafish spleen (a lymphoid organ rich in immune-system cells) and identified *fpr1*-expressing cells (Fig. 6c,d). This suggests that, in zebrafish, *fpr1* does not serve a chemosensory function, but instead fulfills a role in immunity consistent with *FPR1* in humans and mice. It has been previously suggested that the expansion in number of *Fpr* genes to generate vomeronasal chemosensory receptors is specific to the rodent lineage^38^. We identified genomic synteny between zebrafish and mouse at the *Fpr1* locus, but this breaks down around the expanded *Fpr* genes in mouse (Fig. 6e). These are in close proximity to a large cluster of *V2r* genes suggesting their expression in the VNO may be due to a hitchhiking effect^42^, where a duplicated rodent *Fpr* gene fell under the control of a *V2r* enhancer and was co-opted into a new olfactory role. We have therefore demonstrated that zebrafish OM contains all but one of the known types of chemosensory neuron founds in mammals, with the missing class likely to be a neo-functionalization restricted to the rodent lineage.

**Figure 7:**
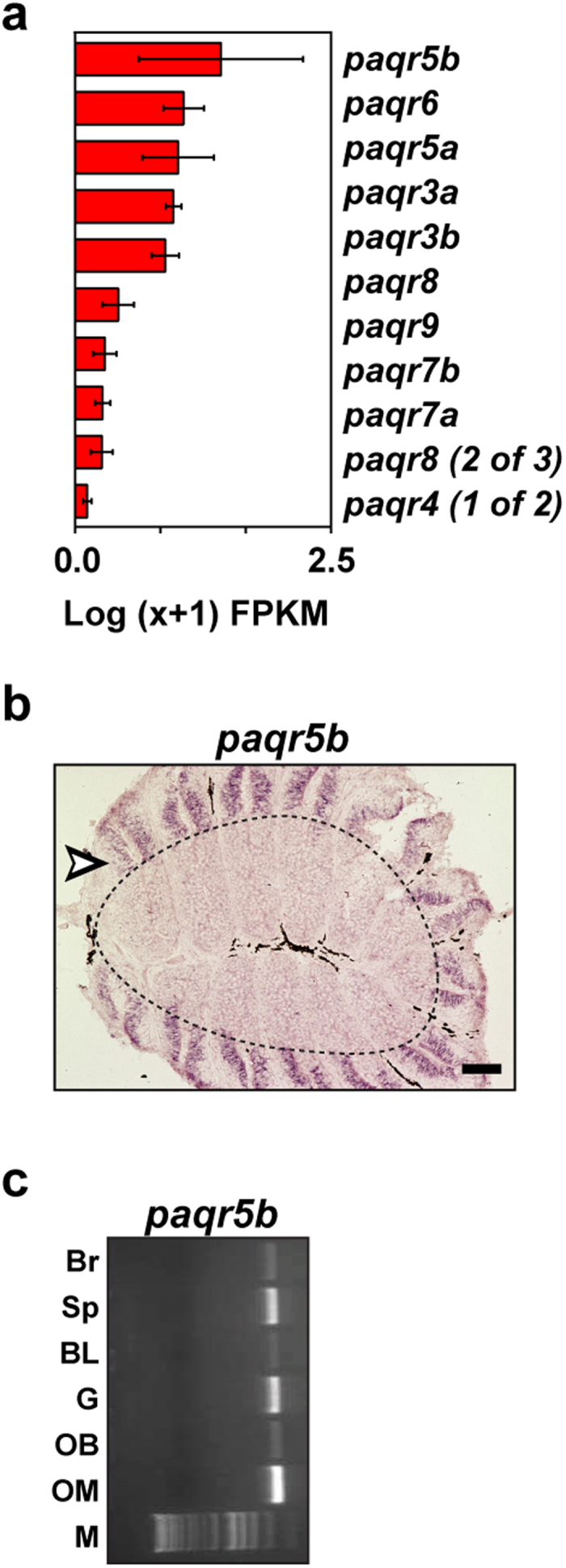
Expression of the *paqr* gene family in the zebrafish OM (**a**) The mean expression values (Log(x+1) FPKM) for all of the annotated *paqr* genes in the zebrafish OM. Genes are sorted in descending order of expression. The error bars represent the standard error of the mean from the 3 biological replicates. (**b**) Cryosections of adult zebrafish OM were hybridized with cRNA probe for *paqr5b*. The sensory neuroepithelium is the area inside the dashed line, while the outside area is the non-sensory region of the zebrafish OM (arrowhead). (**c**) Expression of *paqr5b* mRNA detected by RT-PCR in several tissues. PCR amplifications were performed by using gene-specific primers. OM, olfactory mucosa; OB, olfactory bulb; G, gills; BL, barbels and lips; Sp, spleen; Br, brain. Scale bars, 50 μm.

It has previously been proposed that progestin and adipoQ receptors (*paqr*) *paqr5b* and *paqr6* might serve a chemosensory function in teleost fish, namely in the detection of progestin pheromones^43^. Out of the 11 annotated *paqr* genes in the zebrafish genome, the most abundant is *paqr5b*, with an expression value of 26.53 ± 5.45 FPKM (Fig. 7a). To investigate whether PAQRs are expressed in the sensory region of the OM, we performed ISH with a cRNA probe against *paqr5b*. Surprisingly, *paqr5b* expression is confined to the non-sensory region of the zebrafish OM (Fig. 7b). Moreover, RT-PCR revealed that *paqr5b* is broadly expressed across a range of non-sensory tissues (Fig. 7c). Together these results do not support the hypothesis that *paqr5b* is a pheromone receptor in fish. However, without a complete detailed examination of the remaining *paqr* family members, we cannot exclude the possibility that another *paqr* might be expressed in OSNs, potentially serving a chemosensory function.

## Discussion

We conducted an analysis of the transcriptional profile of the single zebrafish olfactory organ, and compared it to the transcriptomes of the segregated olfactory sub-systems of the mouse: the olfactory mucosa and vomeronasal organ. On a global level, our interspecies comparisons revealed that tissue samples from the whole brain and from the different olfactory organs clustered together first by species and only then by organ (Fig. 4c,d). For the majority of organs the opposite is true, however gene expression in neural tissues (like the brain and cerebellum) has been shown to cluster by species in other studies^44^,^45^,^46^. It has been proposed that neural tissues contain a greater set of genes that are differentially expressed relative to non-neural tissues because of the strong selective pressure acting on the peripheral and central nervous systems to generate adaptive behavior^47^. Indeed, the vertebrate olfactory system is characterized by rapid, species-specific gene gain and losses. This leads to strikingly different gene repertoires reflecting the specific ecological needs of each species^21^,^48^. But how are these differences reflected at the level of gene expression?

Here we found that individual chemosensory receptors are expressed at different levels within three of the four families, with the majority of the receptors expressed at low to moderate levels and some receptors expressed at very high levels (Fig. 1). Overall the exponential-like distributions are very similar within the *or*, *taar* and *olfC/V2r* families, but the distribution of expression in the *ora/V1r* family is less variable. This may be due to the small number of receptors in the *ora* family, or could indicate that they have a more specialized function. This is supported by phylogenetic studies showing that *ora* genes are highly conserved across teleosts and have not undergone the species-specific gene gain and losses characteristic of the other families^8^.

The unusual one receptor-one neuron expression paradigm that is highly prevalent in the olfactory system^17^,^20^,^25^ raised the possibility that the unequal distribution of chemoreceptor expression may act as a proxy for the frequency of neurons expressing each receptor. Alternatively, variance in expression levels per neuron could be the basis of the tissue wide receptor expression profiles we observe. We found that receptor gene FPKM levels correlate with the number of receptor neurons in the zebrafish OM (Fig. 2), suggesting that variance in a receptor’s expression across the neurons in which it is expressed is either negligible, evened out across the population or consistent with the neuronal distribution. We caution that these correlations are extrapolated from counting a limited number of receptor sub-types (5.4%), but note a similar correlation was previously reported between the number of approximately 1% of OSN subtypes and their OR RNA abundances in mouse OM^18^,^49^.

What dictates whether a particular receptor-neuron is highly or poorly represented in the zebrafish OM? Monogenic olfactory receptor selection is still a poorly understood process but, in the mouse, two *cis*-acting regulatory elements have been described as being necessary for the selection of a small number of receptors in their local proximity^16^,^18^,^50^. We could not identify a consistent pattern of expression frequency *vs*. chromosomal location of the receptors, with directly adjacent receptor genes frequently having very different FPKM values (Supplementary Fig. S2). We therefore consider it unlikely that such enhancer elements are sufficient to control a number of different selection probabilities within the same receptor cluster. However, their differential interaction with individual receptor gene promoter sequences could fulfill this role. In mice, the full chemoreceptor expression profiles of both OM and VNO display very similar distributions^51^, suggesting a similar mechanism is likely to operate in sculpting the neuronal distribution in mammalian olfactory organs (with the possible exception of the mouse septal organ, in which one olfactory receptor, SR1 (*Olfr124*), is expressed in a disproportionately large proportion of neurons^52^).

Chemoreceptors from two more families are expressed in sparse sets of neurons in mouse olfactory organs: the membrane-associated guanylate cyclase D (*Gucy2d*) in the OM and five formyl-peptide receptors (*Fpr-rs1, Fpr-rs3, Fpr-rs4, Fpr-rs6*, and *Fpr-rs7*) in the VNO. Our RNA-seq and phylogenetic analysis revealed that guanylate cyclases are also expressed in the zebrafish OM. Also, our ISH experiments showed that *gucy2f –* the zebrafish ortholog of mouse *Gucy2d* – is expressed in a small subset of OSNs scattered around the sensory region of the zebrafish OM (Fig. 5a,b). In the mouse, OSNs that express *Gucy2d* mediate the transmission of preference for food odors via two molecularly distinct ligands, uroguanylin, and carbon disulfide^36^,^37^. In addition, the cyclase domain of the protein can be stimulated by bicarbonate, making *Gucy2d*-expressing OSNs sensitive to carbon dioxide (CO_2_)^35^. Zebrafish are sensitive to low levels of environmental CO_2_^53^, though this is thought to be largely mediated by chemosensitive cells in the gills^54^. Both uroguanylin and another structurally related *Gucy2d* ligand, guanylin, are present in teleost fish^55^, but they also regulate renal and intestinal physiology via other guanylate cyclases^56^. Additional work will therefore be required to determine the precise chemosensory function of *gucy2f*-expressing OSNs in zebrafish.

We were surprised to note that another guanylate cyclase, *gucy1b2* (also known as *CR352256*), was expressed at a higher level than *gucy2f* in the zebrafish OM. The orthologous gene (*Gucy1b2*) had not previously been implicated in having a chemosensory role in mice, but ISH revealed it to pattern small subsets of neurons in both species, similar in number and distribution to those OSNs expressing a specific chemosensory receptor (Fig. 5h). During the revision of this manuscript, *Gucy1b2*-expressing neurons were independently identified in mouse using serial analysis of gene expression^57^. The gene co-patterns a subset of *Trpc2*+ neurons in mouse OM^58^, which also express some (*Omp*, *Cnga2*) but not other (*Adcy3*, *Cnga4*) markers of canonical OSNs. No evidence of canonical chemosensory receptor gene expression was detected by degenerate primer RT-PCR; nevertheless the *Gucy1b2*-expressing neurons project axons to form glomeruli in the olfactory bulb, supporting a chemosensory function^57^. Unlike *Gucy2d*, which spans the plasma membrane and can bind extracellular ligands directly, *Gucy1b2* forms a soluble guanylate cyclase (sGC). Therefore it could represent an olfactory signaling transduction component downstream of a novel chemoreceptor^59^. Alternatively, rodent *Gucy1b2* is directly activated by membrane diffusible nitric oxide (NO)^60^, raising the possibility that the protein has a direct chemosensory function. NO is exhaled in the breath of mammals, and its levels are increased by airway infection and inflammation^61^. Therefore, analogous to *Gucy2d*-expressing neurons that detect CS_2_ in the breath of other mice^62^, detection of exhaled NO by *Gucy1b2* could provide information about the health status of conspecifics. Little is currently known about NO release in aquatic organisms, prohibiting an assessment of a corresponding role for *gucy1b2* in zebrafish OM.

In summary, we sequenced the complete transcriptome of the zebrafish olfactory system and compared it to the major olfactory transcriptomes of mice. For the first time we were able to characterize the zebrafish OM expression profile of all known chemosensory receptor genes, and demonstrate that gene expression levels predict the number of sensory neurons expressing a given chemosensory receptor. We detected conserved and divergent classes of sensory neurons, but show that overall the mouse and zebrafish neural distribution is closely correlated with their chemoreceptor gene repertoire. These studies also permitted the identification of novel cell types in zebrafish and mouse. Taken together we conclude that the basic molecular mechanisms underlying vertebrate olfaction and all but one of the currently known sensory neuron classes that detect odors and pheromones were already present in the MRCA of the teleost and tetrapod lineages.

## Methods

### Ethics statement

Zebrafish and mice were maintained in accordance with UK Home Office regulations, under a project license approved by the Wellcome Trust Sanger Institute Animal Welfare and Ethical Review Body.

### Zebrafish olfactory mucosa RNA extraction and sequencing

Adult male wild type zebrafish (Ab/Tü, 36 weeks old) were anesthetized and decapitated. Olfactory mucosae were dissected out, and frozen on dry ice. Tissue from 4-5 animals was pooled to obtain enough RNA for each sample. Sample replicates I, II and III are pools of tissue originating in 5, 5 and 4 animals, respectively. RNA was then extracted using the RNeasy mini kit (Qiagen). mRNA was prepared for sequencing using the TruSeq RNA sample preparation kit (Illumina) with a selected fragment size of 200–500 bp. The samples were sequenced on the Illumina HiSeq 2500 platform, generating 100 bp paired-end reads.

### RNA-seq data processing and analysis

To analyze the RNA sequencing results we used the iRAP package with the default options^13^. For read mapping and quantifying expression we selected Tophat2 and HTseq2, respectively^63^,^64^. In brief, RNA-Seq reads were aligned to the zebrafish (Danio_rerio.Zv9.73) or mouse (Mus_musculus.GRCm38.74) genome using Tophat2 with 10 threads to align reads, segment length of 20, with Solexa scale for quality values in FASTQ files, no coverage based search for junctions, minimum intron length of 6, and with mate-specific mean and standard deviation extrapolated from each raw data file.

The number of fragments aligned to each gene was counted using the HTSeq2 package with the script *htseq-count*, mode *intersection-nonempty*, id attribute *gene_id*, and not strand specific. Multi-mapped reads were discarded prior to estimating gene expression levels, as they map to multiple locations in the genome and cannot be assigned unambiguously to any gene. To compare the expression values across genes and conditions, raw count data was transformed into fragments per kilobase of transcript per million fragments (FPKM) with the formula:


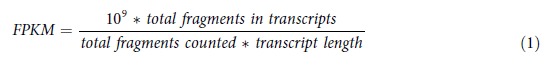


### Data access

RNA-seq data from the zebrafish OM was deposited in the European Nucleotide Archive (ENA) under secondary sample accession numbers: ERS337050, ERS337051, and ERS337052. RNA-seq data for zebrafish brain, mouse OM, mouse VNO and mouse brain were retrieved from the ENA: zebrafish brain (ERR023144, ERR023147, ERR035545), mouse VNO (ERS037281, ERS037283, ERS037286), mouse OM (ERS092547, ERS092549, ERS092545), mouse brain (ERR033015, ERR033016).

### Gene ontology analysis

Gene Ontology Slim (GO Slim) analysis was performed using the WEB-based GEne SeT AnaLysis Toolkit (WebGestalt)^65^,^66^ or Biomart^26^.

### Data mining

All the sequences from annotated and automatically predicted paralogs of *or*, *taar*, *ora/V1r*, *olfC/V2r*, *gucy* and *fpr* genes were extracted from the Ensembl zebrafish genome (Zv 9.0, release 73). In addition, we used Biomart^26^ to retrieve the predicted zebrafish orthologs of mouse *OR*, *Taar*, *V1r*, *V2r*, *Gucy* and *Fpr* genes. To be considered as a putative chemosensory receptor gene for a given family, a triage of the candidates was performed using the position within each chemosensory receptor family clade in a phylogenetic analysis. Using this strategy we identified a total of 135 *or* (12 unannotated), 118 *taar* (24 unannotated), 5 *ora/V1r* (the genomic fragment where *ora2* is located is missing from the current assembly), and 56 *olfC/V2r* (17 unannotated) genes. For the global comparison of the mouse and zebrafish transcriptomes, we used Biomart to retrieve all the zebrafish-mouse orthologs, along with their orthology type and confidence score.

### Phylogenetic analysis

Multiple alignment program for amino acid or nucleotide sequences (MAFFT, version 5.8, http://mafft.cbrc.jp/alignment/server/, accessed October 2013), was employed for multiple protein alignments using the E-INS-i strategy with the default parameters. Phylogenetic trees were constructed using the neighbor-joining method^67^ and the reliability of each tree node was assessed by the bootstrap method with 1,000 replications.

### Statistical analysis

Statistical analyses were done using GraphPad Prism (version 6.04), PAlaeontological STatistics (PAST, version 2.17c, http://folk.uio.no/ohammer/past/, accessed October 2013), and the R statistical package. Data values were standardized and hierarchical clustering analysis was performed using Euclidean distances with Ward’s method. For principal component analysis, the data matrix was standardized and correlation matrixes used to compute the eigenvalues and eigenvectors (components).

### Fitting distributions for the high- and low-expressed genes

The overall distribution of expression values obtained from RNAseq data is bimodal. It has been proposed that such a distribution arises due to the combination of two normal-like distributions of low- and high-expressed genes^14^. Gaussian mixture models can be used to infer the parameters of these underlying distributions. We used the expectation-maximization algorithm provided in the Mixtools Bioconductor package^68^, using all genes with at least one fragment count in one replicate, for the zebrafish OM samples as previously described^51^. The algorithm converged to optimal values and two distributions were fitted. The algorithm reports, for each gene, its probability of being part of either distribution. Based on this, we arbitrarily considered genes to be highly expressed if they had a 0.25 or greater probability of falling in the distribution containing the highly-expressed genes.

### Cloning and *in-situ* hybridization

Adult wild type zebrafish (Ab/Tü, 8–12 months old) were anesthetized with MS-222 (ethyl 3-aminobenzoate, Sigma) and decapitated. Olfactory mucosae and spleen were dissected out, embedded in TissueTek O.C.T. (Sakura), and frozen at −20 °C. Horizontal 8 μm cryosections were thaw-mounted onto Superfrost Plus slide glasses (Thermo).

Adult mice (C57BL/6J, 8 weeks old) were anesthetized, and perfused with 4% paraformaldehyde (PFA). Snouts were dissected out and, post-fixed at 4 °C for 2 hours in 4% PFA, then decalcified for another 72 hours by immersion in a 50:50 mixture of 4% PFA and 0.5 M EDTA at 4 °C. This was followed by immersion in 30% sucrose for 16 hours at 4 °C. The snouts were then embedded in TissueTek O.C.T. compound (Sakura), and frozen at −20 °C. Coronal 12 μm cryosections were thaw mounted onto Superfrost Plus slide glasses (Thermo), dried at 55 °C for 2 hours, and kept at −20 °C until use.

Zebrafish genomic DNA was extracted using standard protocols and used for PCR-mediated cloning. In this study *in situ* probes for the following genes were used: *or101-1* and *or111-6*; *ora3* and *ora5*; *olfCg1* and *olfCq1*; *taar15* and *taar19l*; *gucy2f*, *gucy1b2, gucy2c, fpr1, paqr5b* and *Gucy1b2*. Following are the primers information: *paqr5b*_fw: TTTCAGCAGCATGTCCACTC; *paqr5b*_rv: TCAAACAGGTACGGGTAGGC; *fpr1*_fw: CTCTGTTGCTGAGCTCACCA; *fpr1*_rv: TCAGGATTGACTTGCGCACT; *gucy2f*_fw: TGTAGGCCCCACTAATCCAG; *gucy2f*_rv: GTCATAGGCCTTCGTCAGGA; *gucy1b2*_fw: GTGGATGGAGTCGCTGAACT; *gucy1b2*_rv: TGCCCTCTTTAAGCTGGTTG; *or101-1*_fw: TGAGCGTACGATAGTTATGTGGCGATGTGT; *or101-1*_rv: ATTGCGGAGGGTGTAGATGATGGGGTTGAGCAGGGGT; *or111-6*_fw: AACCCTCTACGGTACACGACT; *or111-6*_rv: GGACGGAATACAGCAAAGCA; *olfCg1*_fw: AGTCAAGCACTTTGGCTGGT; *olfCg1*_rv: CCTCCCAGCACATGAAAACT; *olfCq1*_fw: GAGATCCAGGGACTTCGTGA; *olfCq1*_rv: CCAGGGCATAAACTGCCTTA; *Gucy1b2_fw*: GCTGGACACCATGTACGGAT; *Gucy1b2_rv*:TCCCACGTCTCCTCTCCAAA. Primers for *ora* genes (*ora3* and *ora5*) and *taar* genes (*taar19l* and *taar15*) were as previously described^7^,^8^.

Resulting fragment lengths varied from 78 to 500 bp. All the zebrafish genes were cloned into pDrive (Qiagen, Hilden, Germany) and later confirmed by sequencing. DNA corresponding to the mouse *Gucy1b2* probe-specific region was synthesized and integrated in the pIDT plasmid by IDT (Integrated DNA Technologies, IDT). Templates for *in situ* hybridization antisense probes were derived from the plasmids by PCR, using the same primer sequences, with or without a T3 (TATTAACCCTCACTAAAGGGAA) promoter site attached to the 5′-end. Digoxigenin (DIG) was incorporated into the probes according to the DIG RNA labeling kit supplier protocol (Roche Molecular Biochemicals, Mannheim, Germany). ISH was performed as described in^8^,^41^. Briefly, zebrafish and mouse cryosections were postfixed in 4% paraformaldehyde for 10–15 min at room temperature. Mouse OM cryosections were incubated for 10 min at room temperature with proteinase K (1:800, Roche Molecular Biochemicals) in Tris-EDTA (pH 8.0). Hybridizations were performed overnight at 58–60 °C using standard protocols. For non-fluorescent detection, probes were visualized using anti-DIG primary antibodies coupled to alkaline phosphatase (Roche Molecular Biochemicals) and NBT-BCIP (Roche Molecular Biochemicals). For fluorescent detection, probes were visualized using anti-DIG primary antibodies coupled to horseradish peroxidase (Roche Molecular Biochemicals) and the direct TSA-FITC Kit (Perkin Elmer).

### Quantification of spatial distribution in the zebrafish OM

Spatial coordinates were measured in arbitrary units and normalized, *cf*.^69^,^70^. For laminar height in the olfactory epithelium the distance between the center of the cell soma and the basal border of the epithelial layer was normalized to the distance between basal and apical border of the epithelial layer at the position of the cell to be measured (h_rel_). Thus the range of values is between 0 (Most basal) and 1 (Most apical). Radial distance was measured from the apex of the lamellar ‘curve’ , i.e. closest to the median raphe, to the cell soma center, and normalized to the distance between the central position and the border of the epithelial section (r_rel_). Finally, the cardinal number of sections, normalized to total number of sections (z_rel_) served as the z axis coordinate. Several hundred cells were measured for each marker and spatial coordinate. Distributions are depicted as histograms.

## Additional Information

**Accession codes:** Newly generated RNA-seq data from the zebrafish OM is available in the European Nucleotide Archive (ENA) under secondary sample accession numbers ERS337050, ERS337051, and ERS337052 (Study: PRJEB4464 : Zebrafish_olfactory_transcriptomics).

**How to cite this article**: Saraiva, L. R. *et al.* Molecular and neuronal homology between the olfactory systems of zebrafish and mouse. *Sci. Rep.* **5**, 11487; doi: 10.1038/srep11487 (2015).

## Supplementary Material

Supplementary table and figures

Supplementary Dataset S1

Supplementary Dataset S2
