# Supplementary material for: Molecular and neuronal homology between the olfactory systems of zebrafish and mouse
Source: Sci Rep. 2015 Jun 25;5:11487. doi: 10.1038/srep11487 (PMC4480006; doi:10.1038/srep11487)
Supplement: Supplementary table and figures [file srep11487-s1.doc]

**Title**

Molecular and neuronal homology between the olfactory systems of zebrafish and mouse

**Authors/Affiliations**

Luis R. Saraiva^1,2,4^, Gaurav Ahuja^3,4^, Ivan Ivandic^3^, Adnan S. Syed^3^, John C. Marioni^2,5^, Sigrun I. Korsching^3,5^, and Darren W. Logan^1,5^,*

^1^ Wellcome Trust Sanger Institute, Wellcome Trust Genome Campus, Hinxton-Cambridge, CB10 1SA, United Kingdom

^2^ European Bioinformatics Institute (EMBL-EBI), European Molecular Biology Laboratory, Wellcome Trust Genome Campus, Hinxton-Cambridge, CB10 1SD, United Kingdom

^3^ Institut für Genetik, Universität zu Köln, 50674 Cologne, Germany

^4^ Co-first author

^5^ Co-senior author

* Corresponding author

**Contact**

Corresponding author: Darren W. Logan

Wellcome Trust Sanger Institute,

Wellcome Trust Genome Campus,

Hinxton-Cambridge, CB10 1SA, United Kingdom

Tel: +44 1223 496854

E-mail: [dl5@sanger.ac.uk](mailto:dl5@sanger.ac.uk)

#### Supplementary Information

**
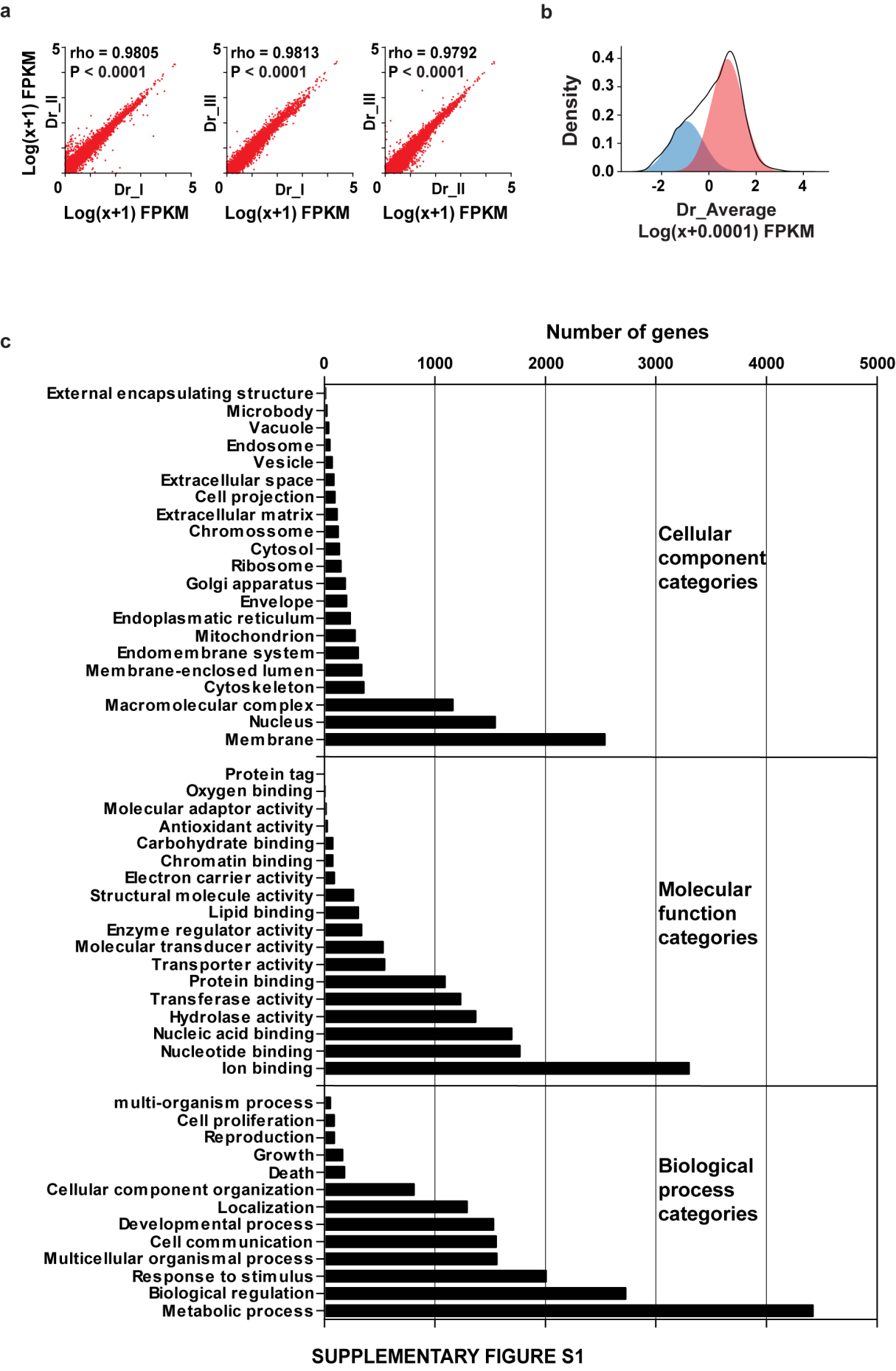
**

**Supplementary Figure** **S1: The transcriptome of the zebrafish OM.** (a) Scatter plots for the expression values for all genes in the three OM biological replicates (I-III). Spearman correlation coefficients were calculated and the rho and p-values indicated for all pairwise comparisons. (b) The zebrafish OM transcriptome shows a bimodal distribution, characteristic of RNA-seq data. Density curves were computed for the mean log_10_ FPKM. To avoid computing Log (0), 0.0001 was added to every value. (c) Gene ontology (GO) analysis for the genes that have a 0.25 or higher probability of being in the highly-expressed distribution in panel B. The number of genes under each category is represented in the x-axis.

**Supplementary Figure** **S2: Expression and chromosomal distribution of the zebrafish chemosensory receptor repertoire.** The mean FPKM expression values are shown for all the chemosensory receptors in the zebrafish OM. Genes are ordered by their chromosomal location and chromosomes are annotated in the boxes at the bottom. *or* genes in red, *taar* genes in green, *ora/V1r* genes in blue, and *olfC/V2r* genes in purple. Error bars represent the standard error of the mean from the three biological replicates.

**
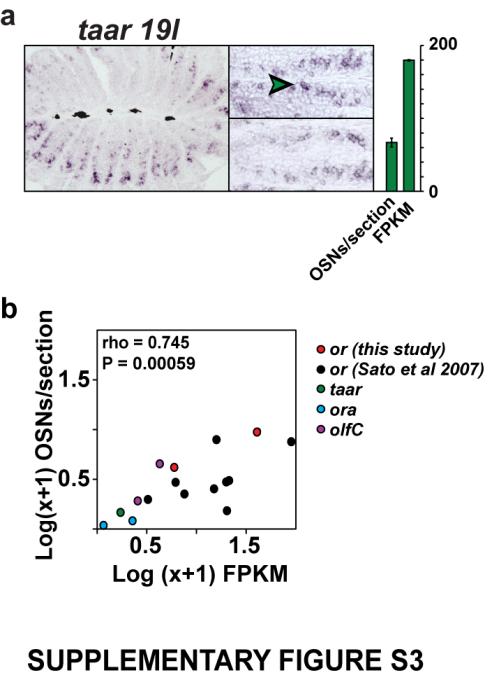
**

**Supplementary Figure** **S3: Comparison of chemosensory receptor gene expression using different methods.**  (a) Cryosections of adult zebrafish olfactory epithelia were hybridized with a complementary RNA (cRNA) probe for *taar19*. Micrographs show expression in complete sections (left panel), and single lamella (right panels). The hybridization signal was observed in somewhat sparse cells within the sensory region of the olfactory epithelia; arrowheads point to single labeled neurons. To the right of the micrograph a bar graph shows number of labeled OSNs/section (mean +/- SEM, 47≤n≤72), and the RNA-seq expression values for *taar19* (mean +/- SEM, n=3). (b) Spearman correlation of FPKM values and OSN density determined by *in-situ* hybridization along with ten additional *or* genes (black dots) retrieved from[^20^](#_ENREF_20). Color code for remaining chemosensory receptor genes is as previously described in Fig. 1.


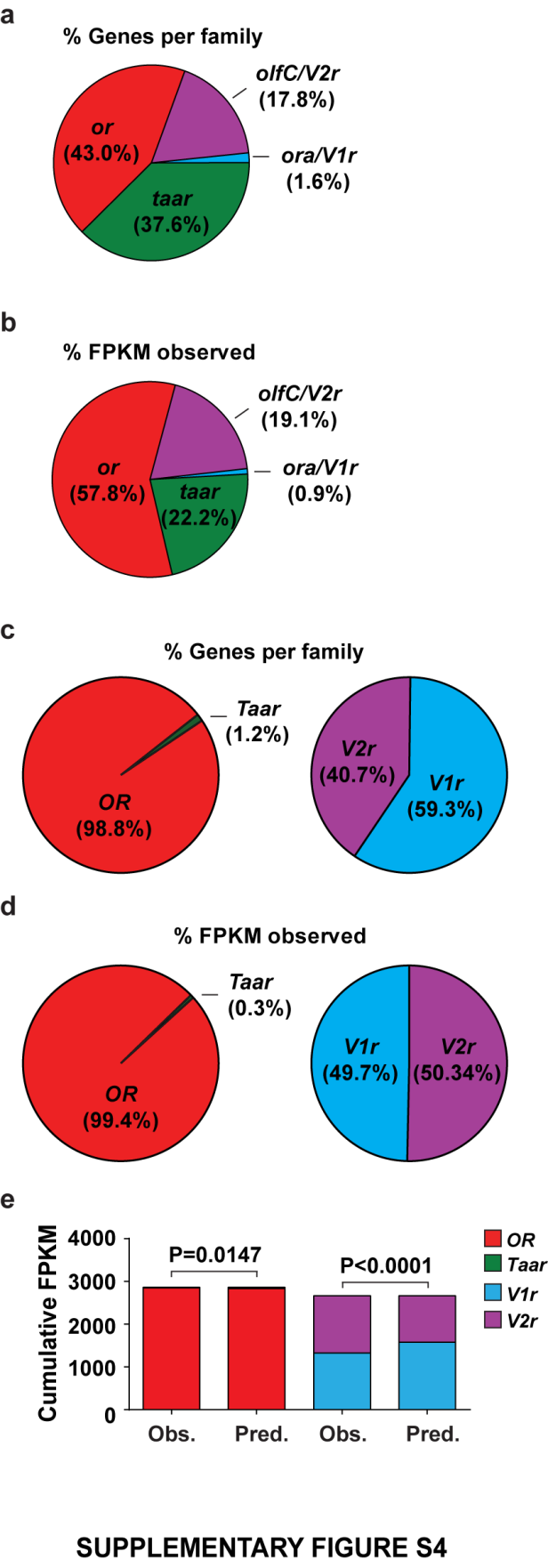


**Supplementary Figure** **S4: Bias in the chemosensory receptor gene expression of mouse and zebrafish.** The relative contribution (%) of each of receptor gene family to the sum of chemosensory receptor genes in zebrafish (a) and mouse (c). The relative contribution (%) of each of receptor gene family to the cumulative expression of chemosensory receptor genes in zebrafish (b) and mouse (d). (e) The observed relative expression of each chemosensory receptor gene family differs from a predicted model where each receptor gene is expressed at the same level in the mouse OM (χ^2^, *P* = 0.0147) and VNO (χ^2^, *P* < 0.0001). *or/OR* genes in red, *taar/Taar* genes in green, *ora/V1r* genes in blue, and *olfC/V2r* genes in purple.

**
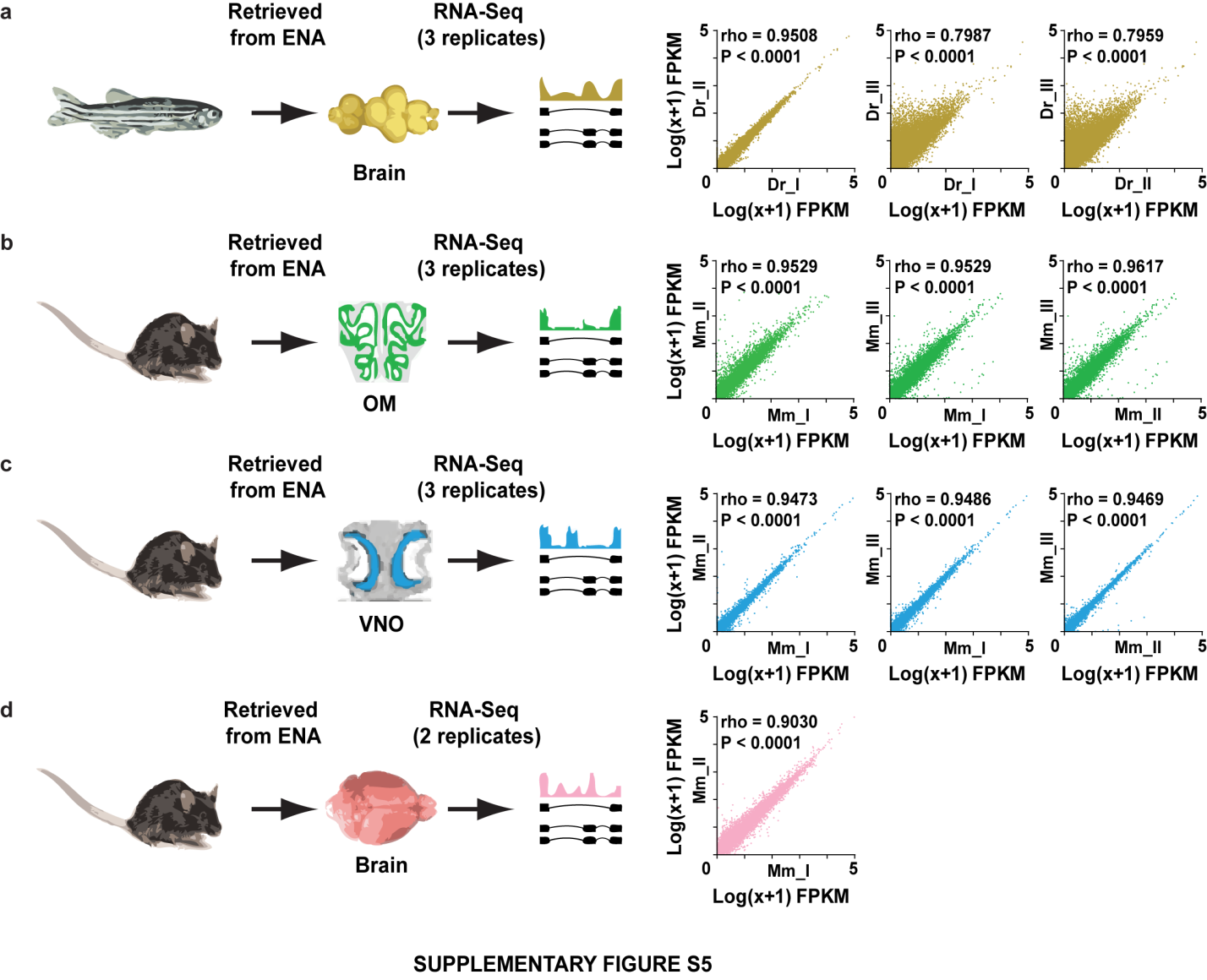
**

**Supplementary Figure** **S5: RNA-seq correlations between biological replicates of the zebrafish brain, and the mouse OM, VNO, and brain.** Scatter plots for the expression values for all genes in the OM, VNO, and brain biological replicates. (a) Zebrafish brain; data for 3 biological replicates retrieved from ENA. Variability amongst the zebrafish brain biological replicates was moderate to extremely low (0.79-0.95, P<0.0001). (b-d) The variability for each mouse tissue revealed extremely low variability levels, as indicated by the high Spearman rho coefficients (0.90-0.96, P<0.0001). (b) Mouse OM; data for 3 biological replicates retrieved from ENA. (C) Mouse VNO; data for 3 biological replicates retrieved from ENA. (d) Mouse brain; data for 2 biological replicates retrieved from ENA). Spearman correlation coefficients (rho) and p-values were calculated and indicated for all pairwise comparisons.


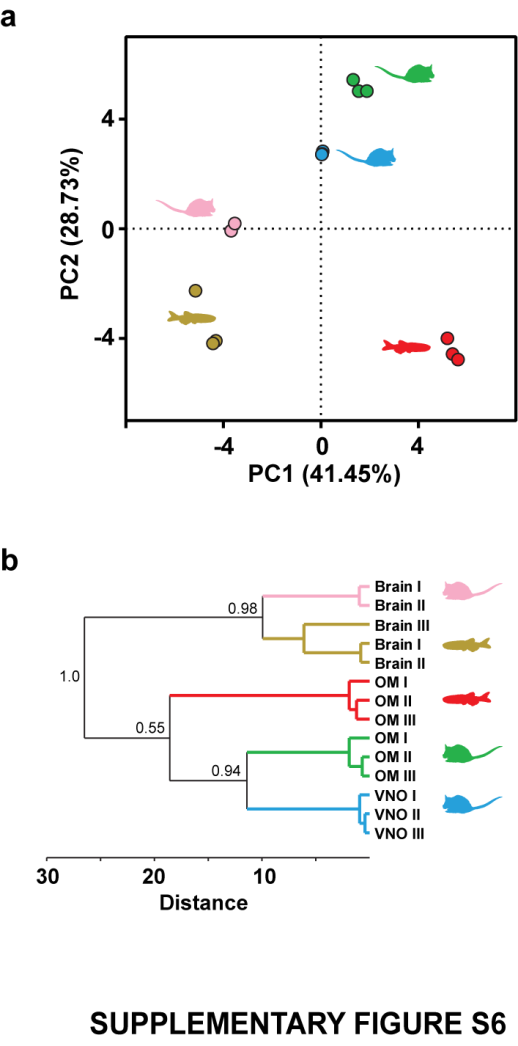


**Supplementary Figure S6: Comparison of the olfactory transcriptomes of zebrafish and mouse.** (a) Principal component analysis (PCA) of the tissue RNA-seq expression levels for the 29 mammalian-zebrafish olfactory cell-specific markers (from Fig. 4e). Percentages of the variance explained by the principal components (PCs) are indicated in parentheses. PC1 separates tissues, while PC2 separates species. (b) Hierarchical clustering analysis (HC) of the tissue expression profiles for the 29 mammalian-zebrafish olfactory cell-specific markers (from Fig. 4e). Bootstrap values (100 boostraps, 1 represents >0.999) for the 3 major nodes are indicated.

**
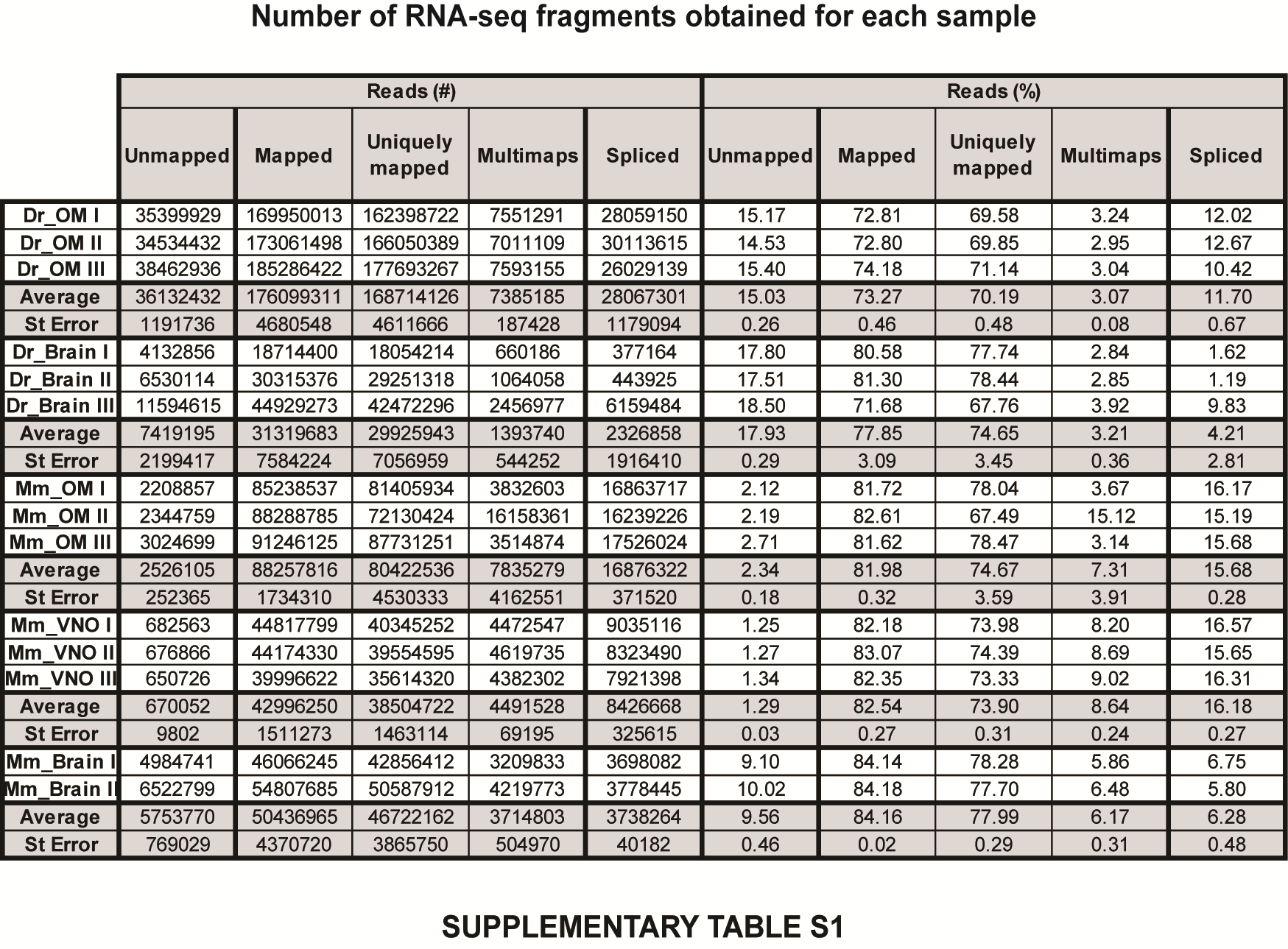
**

**Supplementary Table** **S1: Number of RNA-seq fragments obtained for each sample.** The number and percentages of 100 bp RNA-seq paired-end fragments is indicated for each zebrafish and mouse biological replicates, for all the tissues analyzed.

**Supplementary Data** **S1: Expression estimates in the zebrafish OM.**

A dataset containing the expression values (FPKM) for all the genes in the zebrafish OM.

**Supplementary Data** **S2: Gene accession numbers and expression estimates of all the zebrafish chemosensory receptors used in this study.**

A dataset containing the expression values (FPKM) for all the chemosensory receptor genes in this study.
